# Correlating Eye-Tracking Fixation Metrics and Neuropsychological Assessment after Ischemic Stroke

**DOI:** 10.3390/medicina59081361

**Published:** 2023-07-25

**Authors:** Alec Ionescu, Emanuel Ștefănescu, Ștefan Strilciuc, Alexandru Rafila, Dafin Mureșanu

**Affiliations:** 1 Department of Neuroscience, Iuliu Hatieganu University of Medicine and Pharmacy, 400012 Cluj-Napoca, Romania; ionescu.alec@umfcluj.ro (A.I.); dafinm@ssnn.ro (D.M.); 2 RoNeuro Institute for Neurological Research and Diagnostic, 400364 Cluj-Napoca, Romania; 3 Research Center for Functional Genomics, Biomedicine, and Translational Medicine, Iuliu Hațieganu University of Medicine and Pharmacy, 400012 Cluj-Napoca, Romania; stefan.strilciuc@ssnn.ro; 4 Carol Davila University of Medicine and Pharmacy, Dionisie Lupu Street, 050474 Bucharest, Romania; arafila@yahoo.com

**Keywords:** eye tracking, ischemic stroke, neuropsychological assessment

## Abstract

*Background and Objectives*: Stroke survivors commonly experience cognitive deficits, which significantly impact their quality of life. Integrating modern technologies like eye tracking into cognitive assessments can provide objective and non-intrusive measurements. *Materials and Methods*: This study aimed to evaluate the cognitive and visual processing capabilities of stroke patients using eye-tracking metrics and psychological evaluations. A cohort of 84 ischemic stroke patients from the N-PEP-12 clinical study was selected for secondary analysis, based on the availability of eye-tracking data collected during a visual search task using an adapted Trail Making Test. Standardized cognitive assessments, including the Montreal Cognitive Assessment (MoCA) and digit span tasks, were also conducted. *Results*: Correlation analyses revealed some notable relationships between eye-tracking metrics and cognitive measures, such as a positive correlation between Symbol Search performance and the number of fixations. Anxiety levels were found to be positively correlated with first fixation duration, while longer first fixation durations were associated with poorer cognitive performance. However, most correlations were not statistically significant. Nonparametric ANOVA showed no significant differences in fixation metrics across the visits. *Conclusions*: These findings suggest a complex relationship between cognitive status, gaze fixation behavior, and psychological well-being in stroke patients. Further research with larger sample sizes and analysis of saccadic eye movements is needed to better understand these relationships and inform effective interventions for stroke rehabilitation.

## 1. Introduction

Stroke remains a significant global health burden, affecting millions of people worldwide. It is the second leading cause of death and the third leading cause of disability, showcasing the critical need for comprehensive attention and intervention [1]. In the case of stroke survivors, cognitive deficits are an important aspect alongside motor dysfunction, speech impairment, and additional complications. Despite the fact that the recovery phase often focuses on improvements in physical impairments, cognitive impairment tends to steadily decline, regardless of the initial severity of the stroke. This persistent decline in cognitive function has a substantial impact on the overall quality of life of those affected [2]. An important dilemma in the interconnection between stroke and post-cerebrovascular incident dementia is whether the stroke causes cognitive decline, exacerbates it, or merely brings the issue to the attention of medical professionals. While the question remains unanswered, it is evident that roughly one-third of stroke survivors exhibit significant cognitive impairment within the first few months after the event [3]. While neuropsychological evaluations are routinely used to measure cognitive skills and draw conclusions about real-world capabilities, there is an increasing tendency to integrate modern technologies to enhance these assessments.

Eye tracking (ET) is a technique that provides an objective assessment of both eye movements and the accurate location of one’s gaze [4]. The utilization of eye tracking is increasingly becoming prevalent owing to its capacity to provide improved quantitative parameters for analyzing large data sets. This technique is an accurate, cost-effective, and non-intrusive indicator of cognitive processes [5]. With recent technological advent, eye tracker systems are user-friendly and even portable, demonstrating versatility in various contexts, enabling inquiries that encompass different settings, and facilitating investigations involving diverse demographic groups [6]. The complete potential of this technological advent has yet to be fully realized, and its methods, techniques, and optimal integration of parameters and indicators are still being actively developed [7]. There is a growing interest in eye-tracking parameters as biomarkers. Research has already demonstrated a noteworthy correlation between indicators of oculomotor nerve function and cognition [8]. Eye tracking emerging as a promising diagnostic biomarker for executive function [9].

The efficacy of different saccade paradigms was used in distinguishing patients with mild cognitive impairment (MCI) and Alzheimer’s disease (ADD) from controls. Both prosaccade and antisaccade paradigms successfully differentiated patients from controls. Specifically, antisaccade paradigms demonstrated a greater ability to discriminate patients from controls compared to prosaccade paradigms. Patients with ADD displayed longer prosaccade latencies in the gap and overlap conditions, while patients with ADD also exhibited a higher frequency of antisaccade errors in the gap condition. The gap effect, characterized by a decrease in latency, was consistently observed in both patient groups. Patients with MCI had longer antisaccade latencies than patients with ADD [10]. The investigation of eye movements (EMs) during eye tracking (ET) tasks for Alzheimer’s disease (AD) has identified fixation, smooth pursuit, and saccades as the principal parameters followed [11].

Evaluating cognitive function with standardized testing has always been a matter of debate. For instance, the Mini-Mental State Examination (MMSE), a widely used test for dementia screening, has become less accessible, after its intellectual property rights transfer [12]. A popular alternative cognitive test remains the Trail Making Test. First developed in 1938, as an attention performance test, it was included as a part of the Army Individual Test Battery [13]. The Trail Making Test (TMT) comprises two parts: TMT-A and TMT-B. In TMT-A, participants are asked to connect circles containing numbers from 1 to 25 in an unpredictable arrangement, following the numerical sequence, while completing the task as quickly as possible. TMT-B is similar to TMT-A but requires alternating between numbers and letters. In addition to the direct scores obtained from TMT-A and TMT-B, which measure the completion time for each part, there are other derived scores used, such as the difference score (TMT-B minus TMT-A) and the ratio score (TMT-A divided by TMT-B) [14]. Neurologically, this evaluation activates specific brain regions such as the intraparietal sulcus, right inferior medial frontal cortex, left precentral, medial temporal, angular, and gyrus [15]. TMT is currently tested for the stratification of a normal aging population, people suffering from mild cognitive impairment and dementia [16].

Visual search as a function of cognition has shown potential as a biomarker. A unique visual search behavior pattern is observed in persons diagnosed with behavioral variant frontotemporal dementia (BvFTD). This pattern includes reduced accuracy, longer response times, and an increased frequency and duration of eye movements during visual search tasks [17]. Fixation behavior is influenced by cognitive demand. Eye-tracking studies focused on reading, outlining the link between fixation duration and difficulty while performing cognitive tasks, whereas fixation duration is adapting to processing difficulty while scene viewing [18,19]. Furthermore, recent studies show that the number of fixations reveals attention engagement in young-onset Alzheimer’s Disease [11].

Our research aimed to assess the cognitive and visual processing capabilities of stroke patients, seeking to identify potential correlations between eye tracking and psychological evaluation metrics.

## 2. Materials and Methods

This study is based on secondary data involving a cohort of 84 individuals who had experienced supratentorial ischemic stroke. Evaluated subjects were part of a clinical trial titled “Combined Neuropsychological, Neurophysiological and Psychophysiological Assessment of the Effects of N-Pep-12 on Neurorecovery in Patients After Ischemic Stroke” (protocol available at https://doi.org/10.1186/ISRCTN10702895, accessed on 21 March 2023). Ethical considerations were strictly adhered to throughout the study in accordance with the principles of the Declaration of Helsinki. Prior to their enrollment, all participants provided and signed informed consent. The research protocol underwent review and subsequent approval by the “Iuliu Hatieganu” University of Medicine and Pharmacy Cluj-Napoca, Romania’s Ethics Committee (Approved 10 December 2015 Ethics Committee of the Iuliu Hatieganu University of Medicine and Pharmacy (8 Babeş Street, 400012 Cluj-Napoca, Romania; +40-264-597-256; contact@umfcluj.ro), ref: 507/10.12.2015. Amended twice refs: 82/24.03.2016; 104/12.02.2018).

Our study included participants who met the following inclusion criteria: stroke onset 30–120 days prior to screening; ischemic stroke confirmed by CT or MRI; no significant pre-stroke disability (Modified Rankin Score 0 or 1); Goodglass and Kaplan Communication Scale Score > 2 at screening; no other stroke in preceding 3 months; participant age between 18–80 years, inclusive. Participants were excluded from the study if they met any of the following exclusion criteria: Pre-existing and active major neurological disease; pre-existing and active major psychiatric disease or dementia (IQCODE score > 3); advanced liver, kidney, cardiac, or pulmonary disease; terminal medical diagnosis < 1 year survival; major drug dependency, including alcohol; writing hand injury impacting outcomes; pregnant or lactating females; hemianopsia; neglect; myopia > 3; glaucoma.The characteristics of patients are presented in Table 1.

Evaluations were scheduled for three study visits. A baseline evaluation for all 84 participants was conducted during the initial visit, which took place between 30 to 120 days following the onset of stroke. A trained neurologist clinically evaluated all participants to ensure compliance with the study’s exclusion and inclusion criteria. Subsequent visits were performed at 30 and 90 days after baseline. Our study experienced several dropouts for a variety of factors such as challenges in sustaining follow-up, non-compliance during recording sessions or device calibration difficulty.

**Figure 1:**
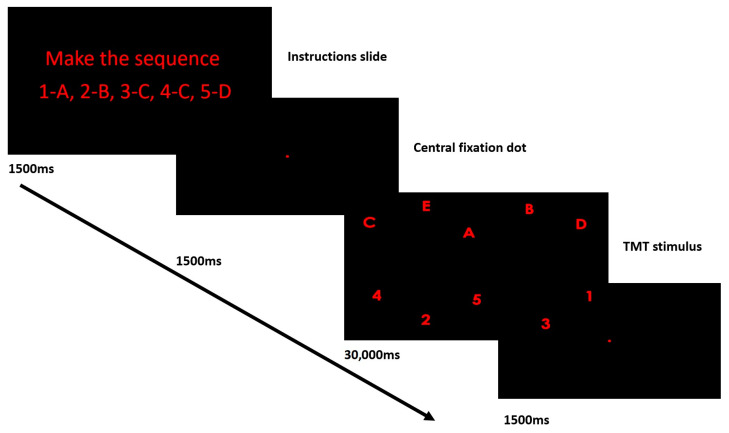
Schematic representation of the experimental protocol.

Tobii Tx300 screen-based, dark pupil, eye tracker (Tobii Technology, Stockholm, Sweden) was used to record eye movements. All recordings were binocular at a sampling rate of 250 Hz. The visual stimulus was presented on a 23-inch display with a 16:9 aspect ratio, featuring a screen resolution of 1920 × 1080 pixels and a refresh rate of 60 Hz. Tobii Studio software was used to generate the visual stimulus, which comprised an instruction slide, a central fixation dot, and an eye-tracking adapted version of the Trail Making Test (TMT). The experimental protocol is depicted in Figure 1. To restrict head motion, a chin and forehead support was used. Participants were seated at a distance of 65 cm from the screen in a room with controlled temperature and dim lighting. Prior to the initiation of each recording, participants underwent a debriefing procedure in which clear task requirements were ensured for an accurate understanding of the experiment. 

In conventional Trail Making Tests, a person has to sequentially connect a given series of letters and/or numbers by using a pencil. In eye-tracking adapted versions of the TMT test the participant is instructed to focus their gaze and perform the specified sequence using saccadic eye movements and fixations. This activity is known as Visual Sequential Search and offers a quantifiable assessment of an individual’s visuospatial capabilities and cognitive function. Participants engaged in a visual sequential search test (VSST) task that required the visual exploration, identification, and completion of the sequence “1-A-2-B-3-C-4-D-5-E.” This sequence needed to be completed at least once throughout the task duration. The task comprised a single trial structured as follows:

Prior to the commencement of recordings, the participants were also provided with a briefing regarding the procedures involved. The instructions were displayed for 1500 milliseconds. Following the instructions, a central dot target was shown for 1500 milliseconds. This stage served to prepare the participants for the upcoming stimulus, by enhancing their attentional focus and priming them for the subsequent stimulus. Subsequently, the Trail Making Test eye-tracking adapted stimulus was displayed for 30,000 milliseconds. The TMT stimulus contained the sequence “1-A-2-B-3-C-4-D-5-E” sequence, which participants had to visually navigate and complete. After the TMT stimulus, the central dot target was displayed again for 1500 milliseconds. This served as a buffer period after the cognitively demanding task. The objective of the task design was to evaluate the visual search ability of the participants and their aptitude to arrange items in a predetermined sequence.

The main focus of this study is fixational parameters throughout a visual search task and exploring their potential as biomarkers of cognitive function. Such parameters can provide information concerning visual exploration, attention distribution, and decision-making processes. By scrutinizing the duration of fixations, one can infer the extent of time participants invest in information processing during the visual task. An analysis of the number of fixations performed by the participant can offer further insights. During the processing of the recordings in Tobii Studio, we established an Area of Interest (AOI) for the Trail Making Test stimulus presentation, the full extent of the screen was taken into account. By ensuring this, every fixation within the screen space while the stimulus was active, regardless of its location, would be captured and included in the analyses.

The following parameters of visual fixation were considered: First Fixation Duration (FFD, measured in seconds). This metric quantifies the duration of the first fixation inside an Area of Interest (AOI). Number of Fixations (NF, i.e., the total count of fixations a participant engages in during the TMT stimulus presentation). Mean Fixation Duration (FDM), which had been previously referred to as Fixation Length in earlier versions of Tobii Studio. This metric measures the duration of each individual fixation within the AOI. Mean Total Fixation Duration (TFDM), encapsulating the aggregate duration of all fixations within an AOI.

The Montreal Cognitive Assessment, commonly referred to as the MoCA, is a useful screening test for detecting cognitive impairment. It assesses key cognitive abilities such as attention, executive functioning, memory, language, visual–spatial reasoning, conceptual thought, computations, and orientation. Anxiety levels were assessed using the Hospital Anxiety and Depression Scale-Anxiety (HADS-A) questionnaire. HADS-A is frequently applied to screen for the prevalence and severity of anxiety symptoms in the community as well as in hospital environments. Hospital Anxiety and Depression Scale-Depression (HADS-D) screened the severity of depressive symptoms. Digit Span Forward (DS-F) was included for assessing working memory, attention, auditory processing, short-term memory, and cognitive processing speed. The DS-F test requires that the individual repeat a series of numbers in the exact same order as the test-giver has presented them. Digit Span Backward (DS-B): Similar to DS-F, this test involves the subject repeating a sequence of numbers, but in the reverse order as presented by the examiner. This task also taps into the working memory component as well as testing the subject’s sequencing capabilities to a greater extent. Processing Speed Index (PSI), more particularly Digit Symbol Coding and Symbol Search, part of Wechsler Adult Intelligence Scale (WAIS-IV) was used to provide valuable information about an individual’s ability to process information efficiently and accurately in a timely manner.

SAS University Edition was utilized to conduct descriptive statistics for each patient visit. We included a variety of eye-tracking parameters: the duration of the initial fixation, the Number of fixations, the mean fixation duration, and the mean total fixation duration. Furthermore, the results of administered psychological assessments were incorporated. Pearson and Spearman correlation analyses were applied to investigate potential relationships between the eye-tracking fixation parameters and the outcomes of the psychological evaluations. A nonparametric One-Way Analysis of Variance (ANOVA) was performed to investigate any variance in the four eye-tracking fixation parameters across the three visits.

## 3. Results

The study included a total of 84 ischemic stroke patients who participated in the evaluation at all three visits. The sample comprised 66 male participants (78.57%) and 18 female participants (21.43%). The age of the participants ranged from 31 to 78 years, with a mean age of 61.5 years (SD = 10.51). Table 2 highlights eye-tracking metrics results for all subjects at baseline.

At baseline, MoCA demonstrated a significant positive correlation with NF during the Trail Making Test (r = 0.19665, *p* = 0.0730) and negative and non-significant correlation with FDM (r = −0.19169, *p* = 0.0807). Symbol Search test results exhibited non-significant correlations with FDM (r = −0.20941, *p* = 0.0559) and a significant positive correlation was observed with NF (r = 0.21964, *p* = 0.0447). The rest of the correlations were non-significant.

At visit 2, the association between DS-F and TFDM reached statistical significance, showing a negative correlation (r = −0.37236, *p* = 0.0031). MoCA scores showed a borderline significant negative correlation with TFDM (r = −0.23644, *p* = 0.0666). For DS-F, a negative correlation with TFDM was statistically significant (r = −0.37085, *p* = 0.0033). DS-B showed a significant negative correlation with TFDM (r = −0.27431, *p* = 0.0324). The rest of the correlations were non-significant.

**Figure 2:**
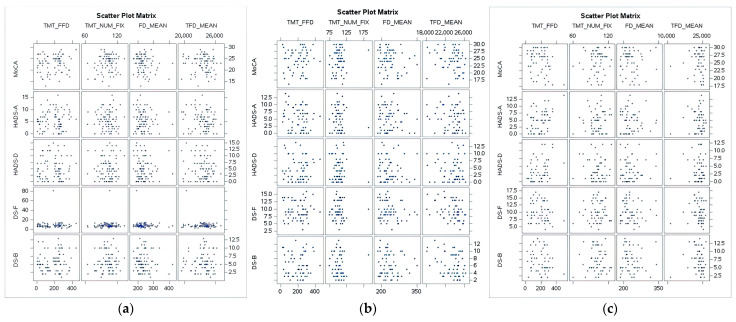
Scatter plot analysis illustrating the relationship between fixation metrics and psychological assessment results at baseline evaluation (**a**) Baseline visit; (**b**) Visit 2; (**c**) Visit 3.

At visit 3, a significant negative correlation was found between MoCA and FFD (r = −0.28792, *p* = 0.0330). Furthermore, the Hospital Anxiety and Depression Scale-Anxiety subscale (HADS-A) showed a significant positive correlation with FFD (r = 0.41412, *p* = 0.0017). For HADS-D, all correlations with eye-tracking metrics were non-significant (FFD: r = 0.26418, *p* = 0.0513). For the HADS-A, a significant positive correlation (spearman) was found with FFD (r = 0.37531, *p* = 0.0048). Apart from this positive correlation, the correlation analysis revealed largely non-significant relationships, as evidenced shown also on scatter plot analysis (Figure 2).

**Figure 3:**
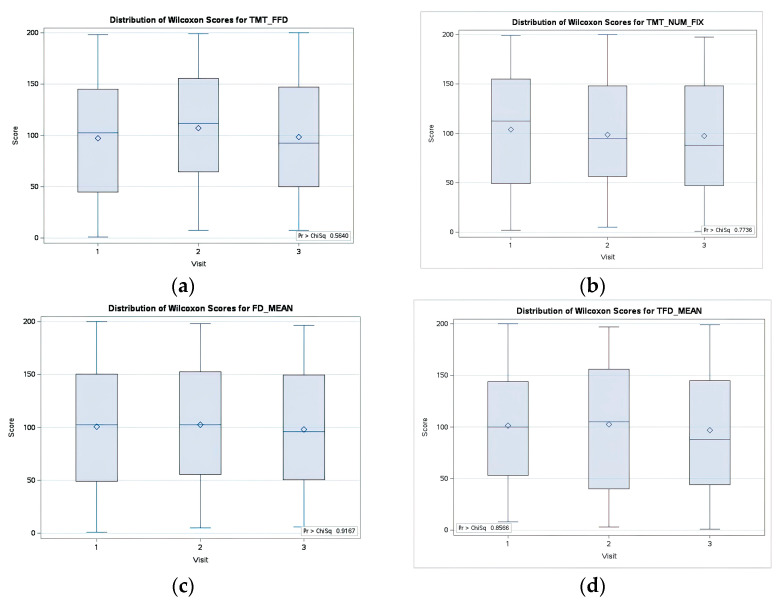
Distribution of Wilcoxon Scores across the 3 visits (**a**) First fixation duration (FFD); (**b**) Number of fixations (NF) (**c**) Fixation duration mean (FDM); (**d**) Total fixation duration mean (TFDM).

A non-parametric one-way ANOVA, using the Kruskal–Wallis H test, was carried out to examine potential statistical differences in eye fixation metrics across three visits. For all parameters, the test yielded a *p*-value > 0.05, thus failing to reject the null hypothesis at the α desired level, indicating no significant variation in FFD, NF, FDM, and TFDM over the three visits (Figure 3).

## 4. Discussion

The primary objective of our study was to evaluate the cognitive and visual processing abilities of individuals who had suffered a stroke. Our aim was to explore the potential associations between eye-tracking fixation metrics and scores obtained from standard psychological evaluations. We gathered parameters of fixation from an eye-tracking-adapted version of the Trail Making Test and a range of psychological evaluations. These included the Montreal Cognitive Assessment (MoCA), the Anxiety and Depression subscales of the Hospital Anxiety and Depression Scale (HADS-A and HADS-D), the forward and backward variants of the Digit Span task, and the Processing Speed Index (Symbol Search and Digit Symbol Coding).

Processing speed, which indicates the speed at which an individual performs mental processes to effectively complete a particular task, is subject to a gradual decline over time [20]. It has been identified as the most solid predictor of an individual’s likelihood to become a SuperAger [21]. Research has shown a correlation between processing speed and the structural integrity of white matter tracts associated with the parietal and temporal cortices, the left middle frontal gyrus, and the superior longitudinal fasciculus [22]. Also, visual processing speed has been linked to a correlation between the inter-FC of the right frontoparietal network (RFPn) and visual networks [23]. Deficits in processing speed constitute a fundamental characteristic of stroke and provide a foundation for post-stroke cognitive impairment [24]. Moreover, processing speed is compromised in aphasia, leading to slowed response times [25]. The number of fixations, or gaze fixation count has been linked to cognitive processing. They reveal the efficiency of visual search or engagement during a cognitive task. A greater frequency of these ocular movements is positively correlated with heightened cognitive load [20]. At baseline evaluation, our analysis of processing speed expressed by Symbol Search showed a positive correlation with the number of fixations made by the subject. This suggests that patients with higher processing speeds may display more gaze fixations [26].

Results from digit span forward and digit span backward, indicators of short-term verbal memory [27], were negatively correlated with the total fixation duration mean. In the context of our visual search task, we observed that prolonged fixation duration is associated with a decline in short-term verbal memory performance. This might suggest that an excessive allocation of cognitive resources toward attention could potentially result in a decline in short-term memory performance. First, the fixation duration refers to the length of time an individual’s eyes remain fixated on a particular object or area during visual processing. It is a measure commonly used in eye-tracking studies to understand visual attention and cognitive processes. Prior studies have suggested that the duration of the first fixation while visually searching typically indicates the characteristics of early processing [28]. Similarly, the first fixation primarily indicates the initial attention allocated to processing the directional characteristics of a stimulus [29].

Our study showed that first fixation duration is positively correlated with anxiety levels, as measured by the HADS-A test. This finding aligns with current research showing that distinct gaze patterns are associated with anxiety symptoms [30]. Specifically, individuals with higher levels of anxiety exhibited prolonged initial attention directed toward the presented stimulus. Moreover, the first fixation duration was also negatively correlated with MoCA scores. This indicates that longer initial attention toward stimulus was associated with poorer cognitive performance at the MoCA assessment. Overall, the majority of the correlations between cognitive and psychological measures and the eye tracking metrics were not statistically significant, with the exception of Digit Span-Forward with TFDM. The lack of significant correlations suggests that these measures may not directly relate to fixational behavior during the Trail Making Test. The nonparametric One-Way Anova evaluation of the eye fixation metrics showed no significant changes across the longitudinal assessment of the post-stroke patients. However, this absence of evidence is not evidence of absence, emphasizing the necessity for additional investigations involving possibly more extensive or diverse participant cohorts. At baseline, the only statistically significant finding was the weak-moderate, positive correlation between the Symbol Search test results and the Number of Fixations. Only negative significant correlations were found at the second evaluation, between Digit Span Forward and Digit Span Backward and TFDM. At the last evaluation, we found a significant positive correlation between Hospital Anxiety and Depression Scale-Anxiety subscale (HADS-A) and FFD. By contrast, FFD is negatively correlated with MoCA scores. The correlation suggests that as anxiety levels (as measured by the HADS-A) increase, so does the FFD. Nonparametric One-Way Anova evaluation showed no significant changes in fixation metrics between visits.

Our study has several limitations. Disease heterogeneity inherent to stroke patients posed a challenge in interpreting our results; in our analysis, we did not account for localization of the lesion, stroke severity, or individual patient characteristics such as sex distribution of the subjects that has been shown to exert a detrimental influence on mortality rates, quality of life, poststroke depression, and activity restrictions, particularly for women [31]. Secondly, our small sample size (n = 84) at baseline and high drop-out rate could have an impact on the generalizability of our results. Lastly, our study did not account for saccadic eye movements during the visual search task or their relationship to fixation metrics. To enhance the reliability and validity of our results, it is recommended that future research include larger sample sizes, a detailed analysis of lesion characteristics, and an integration of saccadic eye movement analysis.

## 5. Conclusions

While our study did identify some correlations between cognitive measures and eye-tracking fixation metrics during the visual search task, these relationships were not consistently robust across different measures and visits. This suggests that while there is a relationship between cognitive status and gaze fixation behavior, there may be a number of other factors at play that this study did not fully account for. A significant implication of this study is that it encourages clinicians and researchers to investigate different approaches that may exhibit a stronger correlation with visuomotor behavior following a stroke. Although cognitive and psychological evaluations offer valuable insights into a patient’s condition, they may not comprehensively account for the variability in visuomotor behavior that is demonstrated during an eye-tracking assessment. Our findings provide a foundation for further investigation into the complex interactions between cognitive status, psychological well-being, and gaze fixation metrics post-stroke. This investigation holds significant importance for clinicians and researchers to design more effective interventions and rehabilitation programs that consider cognitive and visual factors in stroke patients.

## Figures and Tables

**Table 1:** Characteristics of the participants.

	Mean	Median	SD
All patients at baseline visit (V1) *n* = 84 (100%)			
Age at stroke onset	61.5	63	10.50932
Days from stroke	70.15476	67	26.74128
Female *n* = 18 (21%)			
Age at stroke onset	62.27778	63.5	8.456154
Days from stroke	73.16667	75.5	27.36948
Male *n* = 66 (79%)			
Age at stroke onset	61.5	63	10.50932
Days from stroke	70.15476	67	26.74128

**Table 2:** Descriptive statistics of baseline eye tracking parameters.

Visit	N	Variable	Mean	Std Dev	Minimum	Maximum	Median	Range
1	84	First fixation duration (FFD) Number of fixations (NF) Fixation duration mean (FDM) Total fixation duration mean (TFDM)	185.76 101.86 236.16 23,538.32	108.02 13.10 44.55 1637.80	3 64 170 19,443	442 133 422 27,027	212 104 227 23,833	439 69 252 7584
2	61	First fixation duration (FFD) Number of fixations (NF) Fixation duration mean (FDM) Total fixation duration mean (TFDM)	202.65 102.22 235.39 23,383.08	99.00 15.56 37.09 1959.99	20 74 185 18,114.00	455 189 351 26,244	225 102 227 23,882	435 115 166 8130.00
3	57	First fixation duration (FFD) Number of fixations (NF) Fixation duration mean (FDM) Total fixation duration mean (TFDM)	194.42 101.22 231.28 23,120.86	100.83 12.84 35.65 2447.53	20 60 186 11,210	501 125 341 26,379	206 102 225 23,553	481 65 155 15,169

## Data Availability

Data will be deposited into Harvard Dataverse.
